# Characterization of a Novel Chromosomal Class C β-Lactamase, YOC-1, and Comparative Genomics Analysis of a Multidrug Resistance Plasmid in *Yokenella regensburgei* W13

**DOI:** 10.3389/fmicb.2020.02021

**Published:** 2020-08-20

**Authors:** Danying Zhou, Zhewei Sun, Junwan Lu, Hongmao Liu, Wei Lu, Hailong Lin, Xueya Zhang, Qiaoling Li, Wangxiao Zhou, Xinyi Zhu, Haili Xu, Xi Lin, Hailin Zhang, Teng Xu, Kewei Li, Qiyu Bao

**Affiliations:** ^1^The Second Affiliated Hospital and Yuying Children’s Hospital, Wenzhou Medical University, Wenzhou, China; ^2^Key Laboratory of Medical Genetics of Zhejiang Province, Key Laboratory of Laboratory Medicine, Ministry of Education of China, School of Laboratory Medicine and Life Sciences, Wenzhou Medical University, Wenzhou, China; ^3^Institute of Biomedical Informatics, Wenzhou Medical University, Wenzhou, China; ^4^Institute of Translational Medicine, Baotou Central Hospital, Baotou, China

**Keywords:** *Yokenella regensburgei*, β-lactamase, *bla*_*YOC*__–__1_, kinetic analysis, plasmid

## Abstract

*Yokenella regensburgei*, a member of the family Enterobacteriaceae, is usually isolated from environmental samples and generally resistant to early generations of cephalosporins. To characterize the resistance mechanism of *Y. regensburgei* strain W13 isolated from the sewage of an animal farm, whole genome sequencing, comparative genomics analysis and molecular cloning were performed. The results showed that a novel chromosomally encoded class C β-lactamase gene with the ability to confer resistance to β-lactam antibiotics, designated *bla*_*YOC*__–__1_, was identified in the genome of *Y. regensburgei* W13. Kinetic analysis revealed that the β-lactamase YOC-1 has a broad spectrum of substrates, including penicillins, cefazolin, cefoxitin and cefotaxime. The two functionally characterized β-lactamases with the highest amino acid identities to YOC-1 were CDA-1 (71.69%) and CMY-2 (70.65%). The genetic context of the *bla*_*YOC*__–__1_*-ampR*-encoding region was unique compared with the sequences in the NCBI nucleotide database. The plasmid pRYW13-125 of *Y. regensburgei* W13 harbored 11 resistance genes (*bla*_*OXA*__–__10_, *bla*_*LAP*__–__2_, *dfrA14*, *tetA*, *tetR*, *cmlA5*, *floR*, *sul2*, *ant(3″)-IIa*, *arr-2* and *qnrS1*) within an ∼34 kb multidrug resistance region; these genes were all related to mobile genetic elements. The multidrug resistance region of pYRW13-125 shared the highest identities with those of two plasmids from clinical *Klebsiella pneumoniae* isolates, indicating the possibility of horizontal transfer of these resistance genes between bacteria of various origins.

## Introduction

*Yokenella regensburgei* is a species of Enterobacteriaceae originally identified by Kosako et al. (1984) through DNA hybridization and biochemical tests in 1984. In 1985, Hickman-Brenner et al. (1985) proposed the name *Koserella trabulsii* for a new group of Enterobacteriaceae they had discovered that was previously called Enteric Group 45. However, it was subsequently hown by DNA hybridization that *Y. regensburgei* and *K. trabulsii* were the same species (Kosako et al., 1987). In 1991, the Centers for Disease Control and Prevention acknowledged that *Y. regensburgei* had priority upon the basis of prior publication; therefore, the use of *K. trabulsii* was discontinued in 1991 (McWhorter et al., 1991). *Y. regensburgei* has been found in a variety of environmental samples (Jain et al., 2013; Chi et al., 2017). This organism has also been sporadically isolated from clinical samples, thus demonstrating its ability to act as an opportunistic pathogen (Abbott and Janda, 1994; Bhowmick and Weinstein, 2013; Jain et al., 2013; Chi et al., 2017; Palmisano et al., 2019; Wright et al., 2019). Intriguingly, *Y. regensburgei* closely resembles *Hafnia alvei* and thus has possibly been misidentified as the latter by automated systems. It has been hypothesized that infections caused by *Y. regensburgei* were underestimated due to misidentification of the bacterium (Bhowmick and Weinstein, 2013).

Many studies have reported that *Y. regensburgei* possesses an *ampC* gene and that the expression of this gene is highly inducible (Chi et al., 2017). However, to date, there has been no detailed study of the AmpC β-lactamase in *Y. regensburgei*, and no complete genome sequence of *Y. regensburgei* is available in the public NCBI genome database. *Y. regensburgei* appears to be intrinsically resistant to colistin (Hickman-Brenner et al., 1985), azithromycin and some β-lactam antibiotics, and this resistance is likely related to chromosome-mediated AmpC (Jachymek et al., 1999). In addition to the production of ESBLs, resistance to third-generation cephalosporins can also be mediated by chromosomal and plasmid-encoded AmpCs. In general, AmpCs exhibit broad substrate specificity, including penicillins, narrow-spectrum cephalosporins, cephamycins and aztreonam (variable), and their expression can confer resistance to all these compounds. AmpCs are characterized by their greater capability of hydrolyzing cephalosporins and resistance to β-lactam-based β-lactamase inhibitors, such as clavulanate, sulbactam and tazobactam (Jacoby, 2009; Meini et al., 2019).

The acquisition of exogenous resistance genes is often associated with conjugative plasmids, which may carry multiple resistance genes (De Toro et al., 2015; Medaney et al., 2016). Studies have reported that plasmids are abundant in Enterobacteriaceae, such as *Escherichia coli* and *Klebsiella pneumoniae* (Decre et al., 2002; Doi et al., 2002; Gao et al., 2016). However, except for sporadic clinical case reports available in the literature, there have been no reports of the molecular characteristics of the *Y. regensburgei* genome. In this study, for the first time, we determined the complete genome sequence of the *Y. regensburgei* strain W13 isolated from the sewage of an animal farm. Based on sequence analysis, we identified and characterized a novel chromosomally encoded AmpC β-lactamase gene, *bla*_*YOC*__–__1_, and a conjugative plasmid, pYRW13-125, carrying multiple resistance genes. The identification of the novel chromosome-encoded *bla*_*YOC*__–__1_ gene in this environmental bacterium can provide detailed information on the intrinsic resistance mechanism of this unusual opportunistic pathogen.

## Materials and Methods

### Bacterial Strains

*Y. regensburgei* W13 was isolated from sewage discharged from an animal farm in Wenzhou, southeastern China. The strain was first identified using the bioMérieux VITEK 2 Compact Instrument (bioMérieux, Marcy L’etoile, France) and then verified by analysis of the 16S rRNA gene sequences and average nucleotide identity (ANI). The strains and plasmids used in this work are listed in Table 1.

**TABLE 1:** Bacteria and plasmids used in this work.

**Strain or plasmid**	**Relevant characteristic(s)**	**Reference or source**
**Strain**		
W13	The wild-type strain of *Yokenella regensburgei* W13	This study
DH5α	*Escherichia coli* DH5α was used as a host for cloning of the *bla*_*YOC*__–__1_ gene	Our laboratory collection
BL21	*E. coli* BL21 was used as a host for expression of YOC-1	Our laboratory collection
ATCC 25922	*E. coli* ATCC 25922 was used as a quality control for antimicrobial susceptibility testing	Our laboratory collection
pUCP24-*bla*_*YOC*__–__1_/DH5α	DH5α carrying the recombinant plasmid pUCP24-*bla*_*YOC*__–__1_	This study
pCold I-*bla*_*YOC*__–__1_/BL21	BL21 carrying the recombinant plasmid pCold I-*bla*_*YOC*__–__1_	This study
*E. coli* C600	*E. coli* C600 was used as the recipient in the conjugation experiment, RIF^*r*^	Our laboratory collection
**Plasmid**		
pUCP24	Cloning vector for the PCR products of the *bla*_*YOC*__–__1_ gene with its upstream promoter region, GEN^*r*^	Our laboratory collection
pCold I	Expression vector for the PCR products of the ORF of the *bla*_*YOC*__–__1_ gene, AMP^*r*^	Our laboratory collection

**r*, Resistance; RIF, rifampin; GEN, gentamicin; AMP, ampicillin.*

### Antimicrobial Susceptibility Testing

The minimum inhibitory concentrations (MICs) were determined using the agar dilution method following the guidelines of the Clinical and Laboratory Standards Institute (CLSI), and the susceptibility patterns were interpreted according to the CLSI breakpoint criteria (CLSI, 2019). *E. coli* ATCC 25922 was used as a reference strain for quality control.

### Whole-Genome Sequencing (WGS) and Functional Annotation of the Genome Sequence

Bacterial DNA was extracted using the Generay Genomic DNA Miniprep kit (Shanghai Generay Biotech Co., Ltd., Shanghai, China) from a single colony subcultured in brain heart infusion broth at 37°C for approximately 16 h. Genomic DNA was sequenced on a PacBio RS II instrument (Pacific Biosciences). The PacBio long reads were initially assembled by Canu v1.8 (Koren et al., 2017), and then two FASTQ sequence files generated using the Illumina HiSeq 2500 platform were mapped onto the primary assembly to control assembly quality and to correct possible misidentified bases by using Bwa (Li and Durbin, 2009) and the Genome Analysis Toolkit (McKenna et al., 2010). Genes were predicted and annotated by using Prokka v1.14.0 (Seemann, 2014); furthermore, the predicted proteins were searched against the NCBI nonredundant and Swiss-Prot databases using DIAMOND (Buchfink et al., 2015) with an *e*-value threshold of 1e-5. Annotation of the resistance genes was performed using ResFinder (Zankari et al., 2012) and Resistance Gene Identifier (RGI) software of Comprehensive Antibiotic Resistance Database version 4.0.3^1^ (McArthur et al., 2013). GView was used to construct basic genomic features (Petkau et al., 2010). Annotation of MGEs was performed using ISfinder (Siguier et al., 2006) and INTEGRALL (Moura et al., 2009). Average nucleotide identity was calculated using OrthoANI version 0.93.1 (Lee et al., 2016). Easyfig v2.2.2 (Sullivan et al., 2011) software was used to generate the figure showing structural comparisons and the nucleotide identities of the different MDR regions. The molecular weight and pI value of YOC-1 were predicted using ProtParam^2^. The putative signal peptide cleavage site of YOC-1 was identified by SignalP 5.0^3^. Amino acid alignment and neighbor-joining phylogenetic tree construction of YOC-1 with other AmpCs were performed using MEGAX (Kumar et al., 2018).

### Cloning of Bla_*YOC*__–__1_, Expression, and Purification of YOC-1

The *bla*_*YOC*__–__1_ gene sequence with its promoter region and a pair of flanking restriction endonuclease adapters (*Eco*RI for the forward primer pro-*bla*_*YOC*__–__1_-F and *Hind*III for the reverse primer pro-*bla*_*YOC*__–__1_-R, respectively, Table 2) was PCR-amplified, and the PCR product eluted from the agarose gel was digested with the corresponding restriction endonucleases and ligated into pUCP24 (digested with *Eco*RI and *Hind*III) with a T4 DNA ligase cloning kit (Takara Bio Inc., Dalian, China) (Table 2). The resulting recombinant plasmid (pUCP24-pro-*bla*_*YOC*__–__1_) was transformed into *E. coli* DH5α using the calcium chloride method (Suzuki et al., 2019). Transformants (pUCP24-pro-*bla*_*YOC*__–__1_/DH5α) were selected on Luria-Bertani (LB) agar plates containing 40 μg/mL gentamicin. The cloned PCR product was further confirmed by Sanger sequencing (Shanghai Sunny Biotechnology Co., Ltd., Shanghai, China). The resistance activity of the transformants containing *bla*_*YOC*__–__1_ to antibiotics was further determined.

**TABLE 2:** Cloning primers for the *bla*_*YOC*__–__1_ gene.

**Primer^*a*^**	**Sequence (5′–3′)^*b*^**	**Restriction endonuclease**	**Vector**	**Annealing temperature (°C)**	**Amplicon size (bp)**
*orf-bla*_*YOC*__–__1_-F	GGATCCGATGATGATGATAAGGCGCCGAAAACGCTTA	*BamH*I+DDDDK	pCold I	60	1158
*orf-bla*_*YOC*__–__1_-R	AAGCTTTTACTGTAACGCTTTCAGGATGGTGT	*Hin*dIII			
*pro-bla*_*YOC*__–__1_-F	CGGAATTCGATGATATCTGTGCTTAATACAGATT	*Eco*RI	pUCP24	55	1296
*pro-bla*_*YOC*__–__1_-R	CCAAGCTTTTACTGTAACGCGTTATCACACATC	*Hin*dIII			

**^*a*^Primers with “orf” were used to clone the ORF of the bla_*YOC*__–__1_ gene, and primers with “pro” were used to clone the bla_*YOC*__–__1_ gene with its promoter region. ^*b*^The underlined sequences represent the restriction endonuclease sites and their protective bases.**

The predicted cleavage site of the YOC-1 β-lactamase is located after the Ala-22 residue. In order to obtain the β-lactamase YOC-1, the ORF of *bla*_*YOC*__–__1_ without a signal sequence was cloned with a pair of flanking restriction endonuclease adapters (*Bam*HI and DDDDK for the forward primer orf-*bla*_*YOC*__–__1_-F and *Hind*III for the reverse primer orf-*bla*_*YOC*__–__1_-R, Table 2) by similar procedures as described above. Using pCold I as a cloning vector and *E. coli* BL21 as the recipient, transformants (pCold I-*bla*_*YOC*__–__1_/BL21) were selected on LB agar plates containing 100 μg/mL ampicillin. The overnight culture of the recombinant strain (pCold I -*bla*_*YOC*__–__1_/BL21) was diluted 100-fold in 1 L of LB medium, incubated for 2–3 h at 37°C with orbital shaking at 250 rpm until the OD_600_ reached 0.6, and incubated with 0.5 mM isopropyl-β-d-thiogalactopyranoside (IPTG) (Sigma Chemicals Co., St. Louis, MO, United States) for 24 h at 16°C (Choi and Geletu, 2018). The recombinant protein was purified by affinity chromatography using BeyoGold His-tag Purification Resin (Beyotime, Shanghai, China) according to the manufacturer’s instructions. The histidine tag was removed by Enterokinase (GenScript, Nanjing, China) for 24 h at 23°C.

### Conjugation Experiment

*E. coli* C600 (MIC to rifampicin > 2048 μg/mL) was used as the recipient in conjugation experiments to detect the transferability of plasmids carried by *Y. regensburgei* W13 using the broth dilution method. The transconjugant was selected on a brain heart infusion plate supplemented with 2048 μg/mL rifampicin and 16 μg/mL florfenicol. The candidate transconjugant was first tested by plasmid profiling and then indole biochemical property and 16S rRNA gene sequence analyses, which could distinguish whether the candidate transconjugant was the donor, *Y. regensburgei* W13, or the recipient, *E. coli* C600 (*Y. regensburgei* W13 is negative but *E. coli* C600 is positive for the indole test) (Abbott and Janda, 1994). The plasmid of the transconjugant (pYRW13-125/EC600) was further analyzed by PCR and sequencing for the presence of resistance genes.

### Enzyme Kinetic Analysis

Kinetic parameters for hydrolysis of β-lactams by the purified β-lactamase YOC-1 were examined using a UV-VIS spectrophotometer (U-3900, HITACHI, Japan) at 37°C in 10 mM phosphate buffer (pH 7.0) in a final reaction volume of 200 μL. The steady-state kinetic parameters (*k*_*cat*_ and *K*_*m*_) were determined by nonlinear regression of the initial reaction rates with the Michaelis-Menten equation in Prism (version 7) software (GraphPad Software, CA, United States) (Chen et al., 2019).

β-Lactamase inhibition was studied with nitrocefin (100 μM) as the substrate. The β-lactamase inhibitors avibactam, GMP (disodium 5′-guanosine monophosphate) and clavulanic acid at various concentrations were preincubated with purified YOC-1 at 37°C for 5 min before the addition of substrate. The inhibitor concentration required to reduce the hydrolysis of 100 μM nitrocefin by 50% was determined by nonlinear regression with the log (inhibitor) vs. response – Variable slope equation in Prism (version 7) software (GraphPad Software, CA, United States) (Chen et al., 2019).

### Nucleotide Sequence Accession Numbers

The complete chromosome and plasmid sequences of *Y. regensburgei* W13 have been submitted to GenBank, and the accession numbers of the chromosome, pYRW13-125 and pYRW13-131 are CP050811, CP050812, and CP050813, respectively.

## Results and Discussion

### Genome Characteristics and the Resistance Profile of *Y. regensburgei* W13

The 16S rRNA gene similarity analysis showed that *Y. regensburgei* W13 had the closest relationship with *Y. regensburgei* CIP 105435 (NR_104934.1) at 99.00% coverage and 98.17% identity. The average nucleotide identity (ANI) analysis results revealed that W13 shared high identity (99.05%) with *Yokenella regensburgei* ATCC 43003 (NZ_AGCL00000000), and this isolate was finally designated *Y. regensburgei* W13.

**FIGURE 1:**
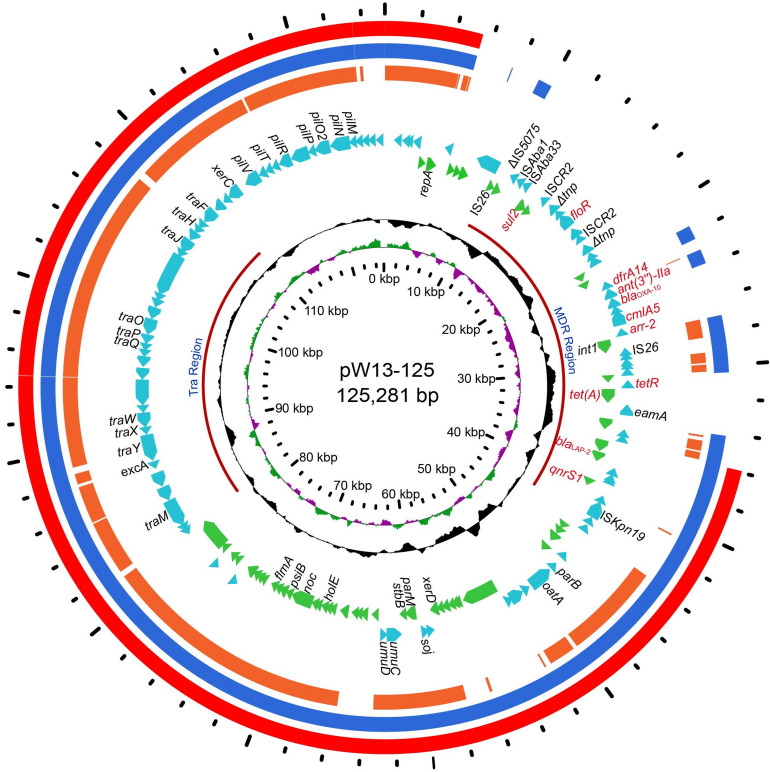
The circular map of pW13-125 and comparative genomics analysis with other closely related plasmids. Circle 1 (from inside to outside) shows the scale in kb. Circles 2 and 3 show the GC skew and GC content, respectively. Circle 4 shows the two different functional regions. Circles 5 and 6 present genes encoded in the forward strand and reverse strand, respectively. Circles 7–9 show the homologous regions of pOP-I (KY126370), an unnamed plasmid (CP027111) and p34399 (CP010385), respectively, and the regions without similar hits were left blank.

The whole genome of *Y. regensburgei* W13 consisted of a chromosome and two circular plasmids. The chromosome was approximately 4.96 Mb in length and encoded 4,802 ORFs. One plasmid, pYRW13-131, was 131,292 bp in length and encoded 184 ORFs, of which 124 were predicted to encode proteins with known functions (Table 3). The other plasmid, pYRW13-125, encoding eleven resistance genes against six classes of antibiotics, was 125,281 bp in length and encoded 150 ORFs (Figure 1). Similar to most *Yokenella regensburgei*, the *in vitro* susceptibility test showed that wild-type *Y. regensburgei* W13 exhibited resistance to penicillin G, ampicillin, cefoxitin, tetracycline and streptomycin (Table 4).

**TABLE 3:** General features of the *Y. regensburgei* W13 genome.

	**Chromosome**	**pYRW13-125**	**pYRW13-131**
Size (bp)	4,962,579	125,281	131,292
GC content (%)	54.40	52.93	47.97
ORFs	4,802	150	184
Known proteins	4,064 (86.63%)	64 (42.67%)	124 (67.39%)
Hypothetical proteins	627 (13.37%)	86 (57.33%)	60 (32.61%)
Protein coding (%)	97.69	100.00	100.00
Average ORF length (bp)	923	717	539
Average protein length (aa)	311	238	179
tRNAs	87	0	0
rRNA operons	(16S-23S-5S)*6	0	0
	(16S-23S-5S-5S)*1		

**TABLE 4:** MICs of 24 antibiotics for 5 strains (μg/mL).

**Antibiotics**	***Y. regensburgei* W13**	**pUCP24-*bla*_*YOC*__–__1_/DH5α**	**pUCP24/DH5α**	**DH5α**	**ATCC 25922**
Penicillin G	128	> 1024	16	16	16
Ampicillin	128	512	4	4	4
Cefazolin	8	256	2	1	1
Cefoxitin	64	128	4	2	2
Ceftazidime	0.25	16	0.06	0.125	0.125
Cefepime	0.06	0.25	0.06	0.125	0.125
Cefoperazone	4	16	0.125	0.125	0.125
Ceftriaxone	0.5	8	0.03	0.03	0.03
Cefotaxime	0.125	0.5	0.03	0.03	0.06
Cefoselis	0.06	0.5	0.03	0.03	0.06
Aztreonam	1	0.125	0.06	0.06	0.25
Imipenem	1	0.5	0.5	0.125	0.125
Florfenicol	256	–	–	4	4
Chloramphenicol	128	–	–	8	4
Nalidixic acid	8	–	–	8	4
Ciprofloxacin	0.03	–	–	0.03	< 0.0075
Tetracycline	128	–	–	2	4
Streptomycin	128	–	–	4	4
Kanamycin	<1	–	–	1	2
Netilmicin	<1			<1	< 1
Levofloxacin	0.5			0.06	0.06
Gentamicin	0.5	–	–	0.25	1
Amikacin	1	–	–	2	1
Tobramycin	0.5	–	–	0.25	0.5

### Homologs to YOC-1 Are Present in Other Enterobacterales Species

**FIGURE 2:**
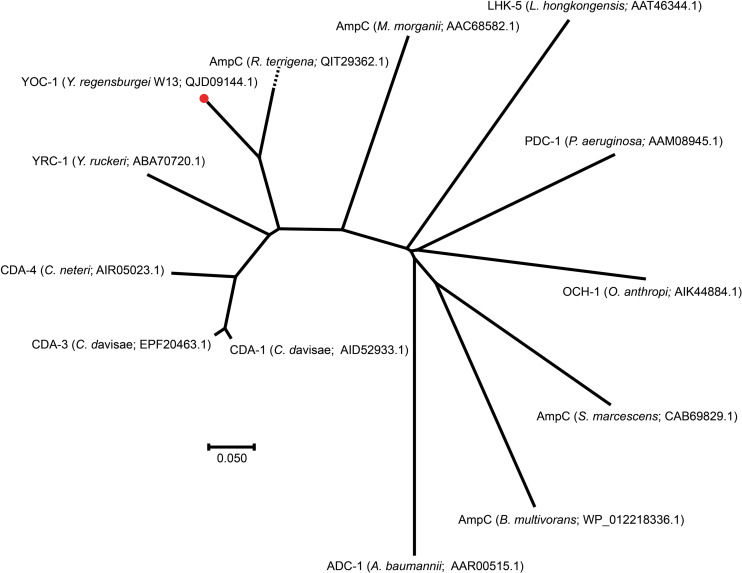
A phylogenetic tree showing the relationship of YOC-1 with other chromosome-borne AmpC β-lactamases. YOC-1 from our study is shown with a red dot.

To investigate whether any novel β-lactamase gene was encoded in the *Y. regensburgei* W13 genome, we analyzed the predicted β-lactamase genes and found one containing conserved motifs of an Ambler class C β-lactamase; the predicted protein shared the highest amino acid identity (71.69%) with a functionally characterized AmpC, CDA-1 (Ammenouche et al., 2014) (Figure 2). We cloned the potential β-lactamase gene, and the results of the antibacterial susceptibility test showed that it was functional (Table 4). The novel β-lactamase gene, designated *bla*_*YOC*__–__1_, is 1,158 bp and encodes a preprotein of 385 amino acids, ca. 41.5 kDa. A signal peptide cleavage site was predicted to be between an alanine and asparagine at amino acid residues 22 and 23, respectively. Furthermore, the pI value of YOC-1 was predicted to be 8.50. Even though a hypothetical class C β-lactamase (EHM51798.1) showing the highest amino acid identity (99.48%, 383/385) with *bla*_*YOC*__–__1_ was predicted to be encoded in a draft genome of *Yokenella regensburgei* ATCC 43003 (NZ_AGCL00000000), the closest functionally characterized relatives to YOC-1 were β-lactamases CDA-1 (AID52933.1) and CMY-2 (X91840.1). They share only 71.69% (274/385) and 70.65% (272/385) amino acid sequence identity with YOC-1, respectively. When searching for YOC-1-like proteins (≥70% aa identity and ≥90% coverage) in the NCBI nucleotide database, it was found that more than half of the close relatives (52.04%, 51/98) were derived from Enterobacter species. YOC-1 had a close relationship with the YOC-1-like AmpCs (Figure 2).

Comparisons of chromosomal (ACT-28 and CDA-1) and plasmidic (CMY-2 and CMY-10) AmpCs that displayed slight carbapenemase activity (Kim et al., 2006; Ammenouche et al., 2014; Kotsakis et al., 2015; Jousset et al., 2019) revealed several conserved residues in CMY-2, CMY-10, CDA-1, and ACT-28 but mutated in YOC-1 [Tyr65, Ala122, Gln231 (Ω loop), Ser244 (Ω loop), Lys274, Leu276, Met278, Asp310 (9 helix), Ala314 (9 helix) and Gly377 (11 helix)] that could lead to the susceptibility of YOC-1 to carbapenem, however, no substitution was observed at amino acid residue 370, which was reported to contribute significantly to the carbapenemase activity of the CMY-2 enzyme^4^. Within the deduced amino acid sequence of YOC-1, three structural elements (one with two copies) characteristic of Ambler class C β-lactamases were identified: S-X-S-K at positions 88–91, K-T-G at positions 339–341, and Y-X-N at positions 174–176 (Supplementary Figure S1). A phylogenetic tree containing YOC-1 and other chromosome-borne class C β-lactamases was constructed (Figure 2), and the results showed that YOC-1 had a close relationship with AmpC derived from *Raoultella terrigena*.

### Functional Characterization of the YOC-1 β-Lactamase

Chromosomal AmpC enzymes, usually inducible, are often expressed at low levels and therefore may not contribute to clinical β-lactam antibiotic resistance (Wu et al., 1999). To assess the potential resistance activities to β-lactams, the coding sequence of *bla*_*YOC*__–__1_ together with its promoter region was cloned into the pUCP24 vector and then transformed into *E. coli* DH5α. The MICs of penicillin G, ampicillin and several first- to fourth-generation cephalosporins against the recombinant strain containing *bla*_*YOC*__–__1_ (pUCP24-*bla*_*YOC*__–__1_/DH5α) increased 4- and 256-fold compared with the control strains (DH5α and DH5α carrying the vector pUCP24). The MIC of benzylpenicillin increased more than 64-fold, the MICs of ceftriaxone, ampicillin and cefoxitin increased at least 256-, 64-, and 32-fold, respectively, and the MICs of cefazolin, ceftazidime and cefoperazone increased 128-fold (Table 4). The results showed that the increase in the MIC of ceftazidime was higher than that of cefotaxime, and ceftazidime is documented to induce AmpC cephalosporinases, which may be the cause of the phenomenon (Jones, 1998). *bla*_*YOC*__–__1_ did not confer resistance to carbapenems, which is different from CDA-1; this result may be due to some mutations in conversed residues associated with the hydrolysis of carbapenem. The antibiotic resistance spectrum of YOC-1 is consistent with most members of the family Enterobacteriaceae with an inducible chromosomal *ampC* gene, which are usually not resistant to extended-spectrum cephalosporins, unless the *ampC* gene is expressed at a very high level. The antibiotic resistance spectrum of YOC-1 is similar to that of LHK-5, a β-lactamase from a clinical *L. hongkongensis* isolate (Lau et al., 2005).

Similar to other chromosome- and plasmid-encoded class C β-lactamases, the catalytic efficiency (*k*_*cat*_/*K*_*m*_) of the purified β-lactamase was high against first- and second-generation cephalosporins, such as cefazolin and cefoxitin, but low against extended-spectrum cephalosporins, such as cefepime (Horii et al., 1993; Raimondi et al., 2001; Lau et al., 2005; Zioga et al., 2008). The highest catalytic efficiency was observed with penicillin G (*k*_*cat*_/*K*_*m*_ rate of 191.5), which is similar to CMY-2 (Kotsakis et al., 2015). YOC-1 was more active against cefoxitin and ceftazidime than CMY-2 because of its much higher *k*_*cat*_, which was partially compensated for by higher *K*_*m*_ values. The turnover rate (*k*_*cat*_) of YOC-1 for ceftazidime was higher than that of CDA-1, which shared 71.69% global amino acid identity with YOC-1 (Ammenouche et al., 2014; Kotsakis et al., 2015). However, the Michaelis constant (*K*_*m*_) of YOC-1 for cefepime was much lower than that of CDA-1. In addition, no measurable hydrolytic activities were observed for aztreonam or imipenem, which is different from CDA-1 (Ammenouche et al., 2014; Table 5).

**TABLE 5:** Kinetic parameters of β-lactam antibiotics for the β-lactamase YOC-1.

**Substrate**	***K*_*m*_ (μM)**	***k*_*cat*_ (s^–1^)**	***k*_*cat*_/*K*_*m*_ (μM^–1^⋅s^–1^)**
Benzylpenicillin	934.20	178900.00	191.50
Ampicillin	109.40	5533.33	50.58
Cefoxitin	33.75	1777.40	52.66
Cefazolin	296.90	16116.20	54.28
Ceftazidime	223.00	20968.89	94.03
Cefepime	341.30	2555.00	7.49
Aztreonam	NH^*a*^	NH^*a*^	NH^*a*^
Meropenem	NH^*a*^	NH^*a*^	NH^*a*^
Imipenem	NH^*a*^	NH^*a*^	NH^*a*^

**Values are the means of three independent measurements. ^*a*^NH, no detectable hydrolysis.**

The hydrolysis of aztreonam was not detectable for the purified enzyme, which was contradicted by the increase in the MIC value of aztreonam against the recombinant carrying *bla*_*YOC*__–__1_, which may have been an experimental error in MIC determination. This discrepancy between phenotypic and biochemical approaches could also result from the low but not zero deacylation rate of AmpC β-lactamases with those compounds. A similar phenomenon was observed for CDA-1, whose host showed resistance to ertapenem, but the β-lactamase did not have hydrolytic activity against ertapenem (Ammenouche et al., 2014).

The results of β-lactamase activity inhibition analysis, as measured by the IC_50_ (50% inhibitory concentration), showed that YOC-1 was poorly inhibited by clavulanic acid (IC_50_: 57.33 ± 0.03 μM) and GMP (IC_50_: 81.73 ± 0.02 μM) and was strongly inhibited by avibactam (IC_50_: 0.001172 ± 0.0002 μM), which is consistent with the majority of class C β-lactamases (Bonnefoy et al., 2004; Na et al., 2017), especially CMY-2 (Bauernfeind et al., 1996; Na et al., 2017), which is the most prevalent AmpC (Doi et al., 2009).

### Comparative Genomic Analysis of the Genetic Context of the Bla_*YOC*__–__1_-Encoding Region

**FIGURE 3:**
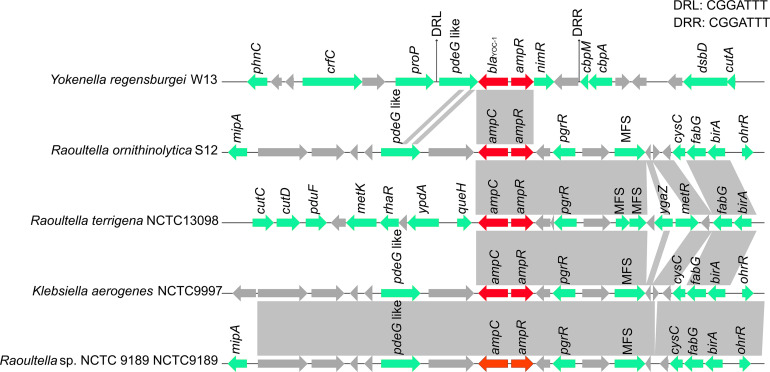
Comparison of the genetic environment of the *bla*_*YOC*__–__1_ gene with those carrying its homologous genes. Genes are shown as arrows and colored based on gene function classification. Genes without functional annotation are colored in gray. Shading denotes homologous regions between them. MFS represents the genes encoding major facilitator superfamily (MFS) transport proteins.

To compare the genetic environments of *bla*_*YOC*__–__1_ in *Y. regensburgei* W13 with those of the closest homologous *ampC*-type genes (identity > 80%), the chromosome sequences of three *Raoultella* spp. strains and one *Klebsiella aerogenes* strain were retrieved from the NCBI nucleotide database. The *ampC*-*ampR* genetic locus was conserved but located in quite different genetic environments, and there were no obvious sequence similarities between the *bla*_*YOC*__–__1_-*ampR*-encoding fragment and each of the other four fragments. Moreover, no sequence showing similarity with the *bla*_*YOC*__–__1_-*ampR*-encoding fragment among the completely sequenced bacterial genomes was retrieved from the NCBI nucleotide database. Furthermore, the region encoding *pdeG*-*bla*_*YOC*__–__1_-*ampR-nimR-hp* was flanked by a pair of perfect 7 bp direct repeats (DRs), indicating that the gene array might be mobile between strains of different species or genera through horizontal gene transfer (HGT). This exclusive gene content of the *bla*_*YOC*__–__1_-*ampR*-encoding region was probably ascribed to the scarcity of completely sequenced genomes of *Yokenella* or other closely related species. Nevertheless, it could not be ruled out that this might be caused by HGT events (Figure 3).

### Comparative Genomics Analysis of the Plasmid Carrying Multiple Resistance Genes

In this study, 11 resistance genes related to different mobile genetic elements were identified in the plasmid pYRW13-125, including two β-lactam resistance genes (*bla*_*OXA*__–__10_ and *bla*_*LAP*__–__2_), two tetracycline resistance genes (*tetA* and *tetR*), one chloramphenicol resistance gene (*cmlA5*), one chloramphenicol/florfenicol resistance gene (*floR*), one sulfanilamide resistance gene (*sul2*), one aminoglycoside resistance gene (*ant(3″)-IIa*), one diaminopyrimidine resistance gene (*dfrA14*), one rifampicin resistance gene (*arr-2*) and one quinolone resistance gene (*qnrS1*) (Figure 1). The plasmid was transferable and could be successfully transferred into recipient cells (*E. coli* C600) through conjugation.

Comparative genome analysis revealed that pYRW13-125 shared the highest identity with three plasmids, including an unnamed plasmid of *Enterobacter hormaechei* BW (CP027111, 81% coverage and 99.98% identity), p34399 of *Enterobacter hormaechei* subsp. *Xiangfangensis* (CP010385, 75% coverage and 100.00% identity) and pOP-I of *Enterobacter cloacae* subsp. *Cloacae* MN201516 (KY126370, 64% coverage and 94.49% identity). The sequence differences among these four plasmids were mainly located in the multidrug resistance (MDR) region (9.70–44.58 kb) of pYRW13-125 (Figure 1).

**FIGURE 4:**
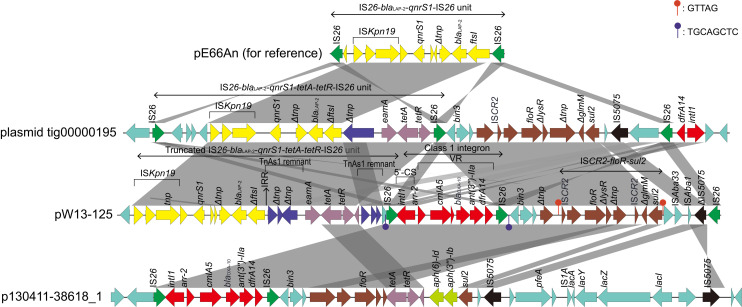
Comparison of the MDR region of pW13-125 with the related regions on the other plasmids. Genes are denoted by arrows and colored according to gene function classification. Shading indicates the homologous regions. The detected direct repeat sequence is marked in the upper right corner.

To better characterize the MDR region, the MDR region of pYRW13-125 was comparatively analyzed with other related sequences. The results showed that the MDR region of pYRW13-125 shared the highest nucleotide sequence similarities with a plasmid from a clinical *K. pneumoniae* strain AR_0152 (CP021946, 99.80% identity and 84% coverage) and a plasmid from the clinical *K. pneumoniae* strain 130411-38618 (MK649826, 99.88% identity and 61% coverage). The MDR region of pYRW13-125 was a complex mosaic structure consisting of 3 mobile genetic element-related units (Figure 4), including a truncated IS*26*-*bla*_*LAP*__–__2_*-qnrS1-tetA-tetR*-IS*26* unit, an atypical class 1 integron, an IS*CR2*-*floR*-*sul2* unit. The plasmid tig00000195 harbors a complete IS*26*-*bla*_*LAP*__–__2_*-qnrS1-tetA-tetR*-IS*26* unit, and the presence of Tn*As1* remnant indicated that this unit might be a result of recombination of two subregions (IS*26*-*bla*_*LAP*__–__2_*-qnrS1*-IS*26* unit and IS*26*-*tetA-tetR*-IS*26* unit) mediated by Tn*As1*. Furthermore, the atypical class 1 integron (organized with a 5′-conserved segment [5′-CS: intI1], a variable region [VR: *arr-2, cmlA5, bla*_*OXA*__–__10_, *ant(3″)-IIa* and *dfrA14*]) without a 3′-CS on pYRW13-125 was identical to that on p130411-38618_1. The integron did not contain the 3′-CS (*qacEΔ1/sul1*) usually found in clinical class 1 integrons, suggesting that this could be a preclinical integron (Ghaly et al., 2017). The integron in pYRW13-125 was immediately flanked by a pair of IS*26*s, suggesting the potential of its mobility. IS*CR2* was found upstream of *floR* and downstream of *sul2* on pYRW13-125. These elements are known to move by a process called rolling-circle replication, and a function of this process is the concomitant movement of accessory sequences found upstream of their transposase genes (Toleman and Walsh, 2010). The presence of IS*CR2* makes the transmission of these two genes possible. The genetic relatedness of the MDR region of pYRW13-125 with those of the two plasmids of clinical strains indicated that the environmental strain W13 might be closely related to multidrug resistant bacteria of human clinical sources.

## Conclusion

In this study, the first complete genome sequence of *Y. regensburgei* was reported. Apart from a novel chromosome-encoded AmpC β-lactamase gene, *bla*_*YOC*__–__1_, the strain also harbored a plasmid (pYRW13-125) encoding multidrug resistance gene arrays that conferred resistance to a series of antibiotics, including tetracycline, amphenicols (chloramphenicol and florfenicol) and streptomycin. Among all of the recently reported functionally characterized β-lactamases, the novel β-lactamase YOC-1 shares the highest amino acid identities of 71.69 and 70.65% with CDA-1 and CMY-2, respectively. The newly identified AmpC β-lactamase encoded in *Y. regensburgei* W13, which carries a multidrug-resistant plasmid, will be helpful for understanding the intrinsic resistance mechanism of this unusual opportunistic pathogen and developing treatments for human (or animal) infections caused by *Y. regensburgei.*

## Data Availability Statement

The datasets presented in this study can be found in online repositories. The names of the repository/repositories and accession number(s) can be found at: https://www.ncbi.nlm.nih.gov/genbank/, MT271603.

## Author Contributions

DZ, ZS, JL, HoL, WL, HaL, XuZ, and QL collected the strains and performed the experiments. DZ, ZS, HX, XL, HZ, TX, and KL analyzed the experimental results and performed the bioinformatics analysis. DZ, ZS, TX, KL, and QB wrote the manuscript. HZ, TX, KL, and QB designed the experiments. All authors read and approved the final manuscript.

## Conflict of Interest

The authors declare that the research was conducted in the absence of any commercial or financial relationships that could be construed as a potential conflict of interest.

## Footnotes

1 https://card.mcmaster.ca/

2 https://web.expasy.org/protparam/

3 www.cbs.dtu.dk/services/SignalP/

4 http://dx.doi.org/10.1111/1574-6968.12199

## Supplementary Material

FIGURE S1 Amino acid alignment of YOC-1 with carbapenem-resistant AmpCs. Dots indicate the absence of amino acid residues. The accession numbers of the cephalosporinases (from up to down) are as follows: QJD09144.1, AID52933.1, AIM49619.1, AAK59969.1, WP_023331841.1. The logo size indicates the degree of conservation of the amino acid residues. The amino acids in proteins with ≥80% identity are shaded in blue. The length of each AmpC protein is marked at the end of the alignment. Conserved motifs are indicated at the top of the sequence alignment. The red star indicates the unique residue in YOC-1, and the predicted signal sequence cleavage site for YOC-1 is indicated by a vertical arrow.
