# Supplementary material for: Characterization of a Novel Chromosomal Class C β-Lactamase, YOC-1, and Comparative Genomics Analysis of a Multidrug Resistance Plasmid in Yokenella regensburgei W13
Source: Front Microbiol. 2020 Aug 20;11:2021. doi: 10.3389/fmicb.2020.02021 (PMC7468467; doi:10.3389/fmicb.2020.02021)
Supplement: FIGURE S1 — Amino acid alignment of YOC-1 with carbapenem-resistant AmpCs. Dots indicate the absence of amino acid residues. The accession numbers of the cephalosporinases (from up to down) are as follows: QJD09144.1, AID52933.1, AIM49619.1, AAK59969.1, WP_023331841.1. The logo size indicates the degree of conservation of the amino acid residues. The amino acids in proteins with ≥80% identity are shaded in blue. The length of each AmpC protein is marked at the end of the alignment. Conserved motifs are indicated at the top of the sequence alignment. The red star indicates the unique residue in YOC-1, and the predicted signal sequence cleavage site for YOC-1 is indicated by a vertical arrow. [file Data_Sheet_1.PDF]

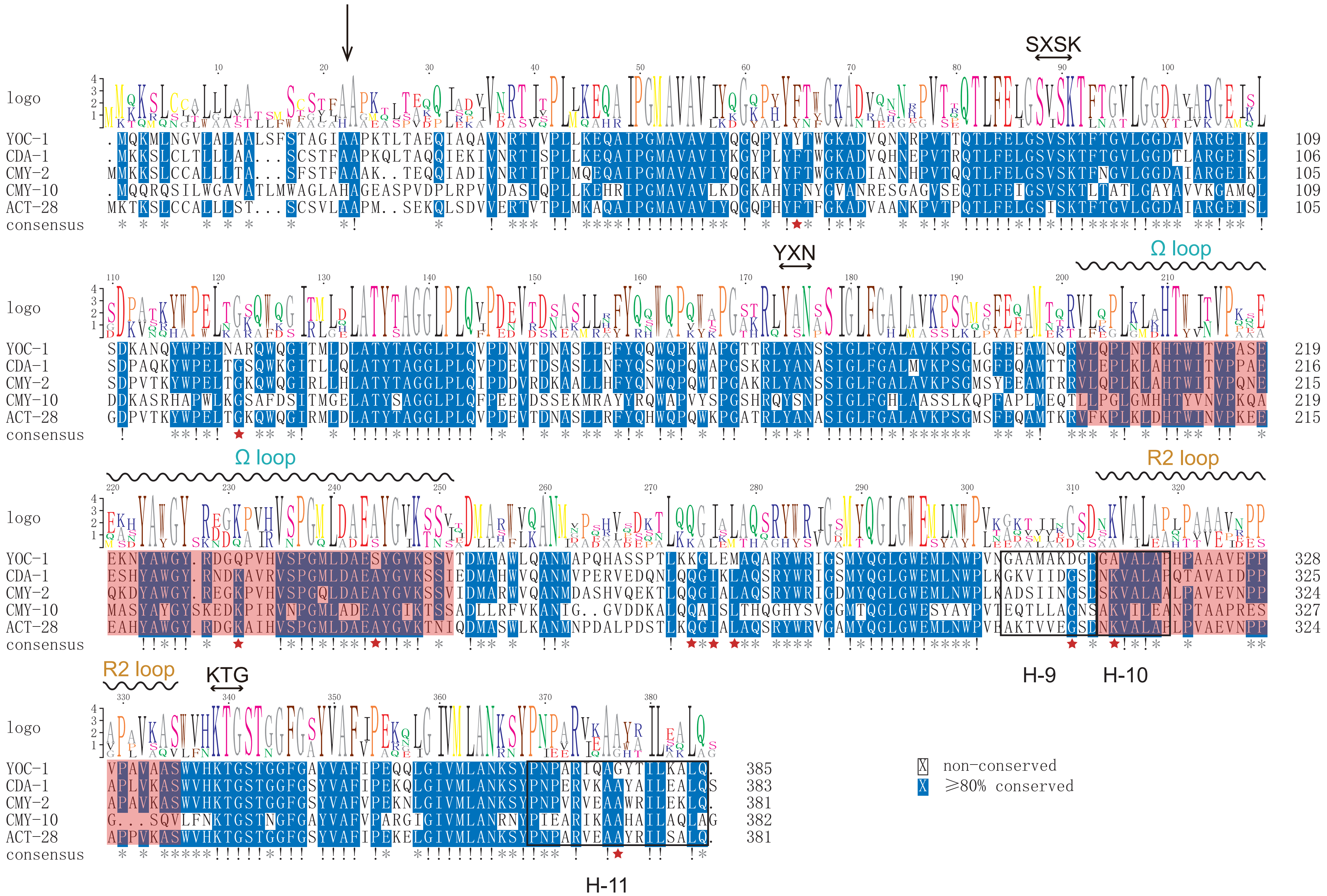
SXSK
logo
YOC-1 .MQKMLNGVLALAALSFSTAGIAAPKTLTAEQIAQAVNRTIVPLLKEQAIPGMAVAVIYQGQPYYYTWGKADVQNNRPVTTQTLFELGSVSKTFTGVLGGDAVARGEIKL 109
CDA-1 .MKKSLCLTLLLAA...SCSTFAAPKQLTAQQIEKIVNRTISPLLKEQAIPGMAVAVIYKGYPLYFTWGKADVQHNEPVTRQTLFELGSVSKTFTGVLGGDTLARGEISL 106
CMY-2 MMKKSLCCALLLTA...SFSTFAAAK..TEQQIADIVNRTITPLMQEQAIPGMAVAVIYQGKPYYFTWGKADIANNHPVTQQTLFELGSVSKTFNGVLGGDAIARGEIKL 105
CMY-10 .MQQRQSILWGAVATLMWAGLAHAGEASPVDPLRPVVDASIQPLLKEHRIPGMAVAVLKDGKAHYFNYGVANRESGAGVSEQTLFEIGSVSKTLTATLGAYAVVKGAMQL 109
ACT-28 MKTKSLCCALLLST...SCSVLAAPM..SEKQLSDVVERTVTPLMKAQAIPGMAVAVIYQGQPHYFTFGKADVAANKPVTPQTLFELGSISKTFTGVLGGDAIARGEISL 105
consensus
YXN
Ω loop
logo
YOC-1 SDKANQYWPELNARQWQGITMLDLATYTAGGLPLQVPDNVTDNASLLEFYQQWQPKWAPGTTRLYANSSIGLFGALAVKPSGLGFEEAMNQRVLQPLNLKHTWITVPASE 219
CDA-1 SDPAQKYWPELTGSQWKGITLLQLATYTAGGLPLQVPDEVTDSASLLNFYQSWQPQWAPGSKRLYANASIGLFGALMVKPSGMGFEQAMTTRVLEPLKLAHTWITVPPAE 216
CMY-2 SDPVTKYWPELTGKQWQGIRLLHLATYTAGGLPLQIPDDVRDKAALLHFYQNWQPQWTPGAKRLYANSSIGLFGALAVKPSGMSYEEAMTRRVLQPLKLAHTWITVPQNE 215
CMY-10 DDKASRHAPWLKGSAFDSITMGELATYSAGGLPLQFPEEVDSSEKMRAYYRQWAPVYSPGSHRQYSNPSIGLFGHLAASSLKQPFAPLMEQTLLPGLGMHHTYVNVPKQA 219
ACT-28 GDPVTKYWPELTGKQWQGIRMLDLATYTAGGLPLQVPDEVTDNASLLRFYQHWQPQWKPGATRLYANASIGLFGALAVKPSGMSFEQAMTKRVFKPLKLDHTWINVPKEE 215
consensus
Ω loop
R2 loop
logo
YOC-1 EKNYAWGY.RDGQPVHVSPGMLDAESYGVKSSVTDMAAWLQANMAPQHASSPTLKKGLEMAQARYWRIGSMYQGLGWEMLNWPVNGAAMAKDGDGAVALAHPAAAVEPP 328
CDA-1 ESHYAWGY.RNDKAVRVSPGMLDAEAYGVKSSIEDMAHWVQANMVPERVEDQNLQQGIKLAQSRYWRIGSMYQGLGWEMLNWPLKGKVIIDGSDNKVALAPQTAVAIDPP 325
CMY-2 QKDYAWGY.REGKPVHVSPGQLDAEAYGVKSSVIDMARWVQANMDASHVQEKTLQQGIALAQSRYWRIGDMYQGLGWEMLNWPLKADSIINGSDSKVALAALPAVEVNPP 324
CMY-10 MASYAYGYSKEDKPIRVNPGMLADEAYGIKTSSADLLRFVKANIG..GVDDKALQQAISLTHQGHYSVGGMTQGLGWESYAYPVTEQTLLAGNSAKVILEANPTAAPRES 327
ACT-28 EAHYAWGY.RDGKAIHVSPGMLDAEAYGVKTNIQDMASWLKANMNPDALPDSTLKQGIALAQSRYWRVGAMYQGLGWEMLNWPVEAKTVVEGSDNKVALAPLPVAEVNPP 324
consensus
H-9
H-10
R2 loop
KTG
logo
YOC-1 VPAVAASWVHKTGSTGGFGAYVAFIPEQQLGIVMLANKSYPNPARIQAGYTILKALQ. 385
CDA-1 APLVKASWVHKTGSTGGFGSYVAFIPEKQLGIVMLANKSYPNPERVKAAYAILEALQS 383
CMY-2 APAVKASWVHKTGSTGGFGSYVAFVPEKNLGIVMLANKSYPNPVRVEAAWRILEKLQ. 381
CMY-10 G...SQVLFNKTGSTNGFGAYVAFVPARGIGIVMLANRNYPIEARIKAAHAILAQLAG 382
ACT-28 APPVKASWVHKTGSTGGFGSYVAFIPEKELGIVMLANKSYPNPARVEAAYRILSALQ. 381
consensus
H-11
X non-conserved
X ≥80% conserved
